# Intraoperative Lung Ultrasound (ILU) for the Assessment of Pulmonary Nodules

**DOI:** 10.3390/diagnostics11091691

**Published:** 2021-09-16

**Authors:** Marco Taurchini, Carla Maria Irene Quarato, Elisabetta Maria Frongillo, Gian Maria Ferretti, Cristiana Cipriani, Marco Bizzarri, Maria Pia Foschino Barbaro, Donato Lacedonia, Annalisa Simeone, Paolo Graziano, Lucia Dimitri, Evaristo Maiello, Lucio Cavaliere, Salvatore De Cosmo, Marco Sperandeo

**Affiliations:** 1 Unit of Thoracic Surgery, IRCCS Fondazione “Casa Sollievo della Sofferenza”, 71013 San Giovanni Rotondo, Italy; m.taurchini@operapadrepio.it (M.T.); marco.bizzarri@inwind.it (M.B.); 2 Department of Medical and Surgical Sciences, Institute of Respiratory Diseases, Policlinico Universitario “Riuniti” di Foggia, University of Foggia, 71122 Foggia, Italy; carlamariairene.quarato@gmail.com (C.M.I.Q.); mariapia.foschino@unifg.it (M.P.F.B.); donato.lacedonia@unifg.it (D.L.); 3 Unit of Thoracic Surgery, Cliniche Humanitas Gavazzeni, 24125 Bergamo, Italy; elisabettamaria.frongillo@gmail.com; 4 Department of Internal Medicine and Medical Disciplines, Sapienza University, 00161 Rome, Italy; cristiana.cipriani@gmail.com; 5 Unit of Radiology, IRCCS Fondazione “Casa Sollievo della Sofferenza”, 71013 San Giovanni Rotondo, Italy; a.simeone@operapadrepio.it; 6 Unit of Pathology, IRCCS Fondazione “Casa Sollievo della Sofferenza”, 71013 San Giovanni Rotondo, Italy; p.graziano@operapadrepio.it (P.G.); l.dimitri@operapadrepio.it (L.D.); 7 Unit of Oncology, IRCCS Fondazione “Casa Sollievo della Sofferenza”, 71013 San Giovanni Rotondo, Italy; e.maiello@operapadrepio.it; 8 Unit of Reanimation and Anesthesiology, IRCCS Fondazione “Casa Sollievo della Sofferenza”, 71013 San Giovanni Rotondo, Italy; cabalucio@gmail.com; 9 Department of Internal Medicine, IRCCS Fondazione “Casa Sollievo della Sofferenza”, 71013 San Giovanni Rotondo, Italy; s.decosmo@operapadrepio.it; 10 Unit of Interventional and Diagnostic Ultrasound of Internal Medicine, IRCCS Fondazione “Casa Sollievo della Sofferenza”, 71013 San Giovanni Rotondo, Italy; sperandeomar@gmail.com

**Keywords:** ultrasound, intraoperative ultrasound, transthoracic ultrasound, thoracic surgery, lung nodules

## Abstract

Background: The primary aim of this study was to confirm the validity of intraoperative lung ultrasound (ILU) as a safe and effective method of localization for difficult to visualize pulmonary nodules during Video-Assisted Thoracoscopic Surgery (VATS) and open thoracotomy. The secondary aim was to enhance knowledge on the morphological patterns of presentation of pulmonary nodules on direct ultrasound examination. Materials and methods: 131 patients with lung nodule and indication for surgery were enrolled. All patients underwent pre-operative imaging of the chest, including Chest Computed Tomography (CT) and Transthoracic Ultrasound (TUS), and surgical procedures for histological assessment of pulmonary nodules (VATS or open thoracotomy). Results: The identification of 100.00% of lung nodules was allowed by ILU, while the detection rate of digital palpation was 94.66%. It was not possible to associate any specific ILU echostructural pattern to both benign or malignant lesions. However, the actual histological margins of the lesions in the operating samples were corresponding to those visualized at ILU in 125/131 (95.42%) cases. No complications have been reported with ILU employment. Conclusions: In our experience, ILU performed during both open surgery and VATS demonstrated to be a reliable and safe method for visualization and localization of pulmonary nodules non previously assessed on digital palpation. In addition, ILU showed to allow a clear nodule’s margins’ definition matching, in most cases, with the actual histological margins.

## 1. Introduction

Pulmonary nodules are commonly observed in clinical practice, with many cases being identified as incidental findings on chest X-ray (CXR) or computed tomography (CT). In the last decades, several guidelines and recommendations have been developed with the aim of identifying, classifying, studying and defining the criteria for surgery or follow-up by chest imaging of lung nodules [1,2,3]. In particular, surgical excision is indicated when malignancy is highly suspected on pre-operative studies. Both Video-Assisted Thoracoscopic Surgery (VATS) and open thoracotomy may be employed. Advantages of VATS over the open approach are reduction of post-operative pain and functional impairment, and therefore of complications, morbidity and mortality [4,5]. Conversely, palpation of the lung surface for localization of non-subpleural lung nodules may be impaired during VATS. This may happen especially in metastatic disease, where the open approach is usually preferred [6]. The use of additional techniques, together with the VATS and the open surgery approach, may help in improving the visualization of the lesions/s [7,8,9]. 

Nowadays, lung ultrasound by transthoracic examination (TUS) has gained a relevant role in the evaluation of lung and pleural diseases [10]. In particular, TUS is a known valid diagnostic support both pre-operatively, for the detection of pleural and subpleural lesions, and during interventional procedures, in guiding diagnostic percutaneous needle biopsies [11,12]. Intraoperative lung ultrasound (ILU) is a novel technique that can be employed for the study of lung and pleural diseases during thoracic surgery. Despite ILU not being routinely performed in clinical practice, encouraging results have been highlighted by early research studies [13,14,15]. The application of ILU during VATS (VATS-US) has been proved to be a simple and safe real-time methodology for intraoperative visualization of pulmonary nodules in a preliminary report from our group [13]. As a further confirmation, ILU was shown to be able to localize lung nodules not identified by digital palpation also in other available experiences [14,16]. 

On this background, the employment of ILU during both VATS and open surgery for the assessment of lung nodules was systematically analyzed in the present study. The primary aim was to confirm the validity of ILU as a localization method for difficult to visualize pulmonary nodules during surgical procedures. The secondary aim was to enhance knowledge on the morphological patterns of presentation of pulmonary nodules on direct ultrasound examination (i.e., during surgery). In this perspective, data derived from pre-operative radiological exams, pre-operative TUS images, ILU images obtained during surgery and histological examination were reported. The characteristics of the nodules at ILU were therefore correlated with their histological benign or malignant nature and compared with the characteristics presented at the pre-operative TUS examination.

## 2. Materials and Methods

This is an observational prospective study whose primary aim was to confirm the validity of ILU as a safe and effective localization method for difficult to visualize pulmonary nodules during VATS and open surgery. In addition, ILU effectiveness in allowing the morphological characterization of lung nodules according to their histological benign or malignant nature was analyzed, including also a comparison with the results obtained from the pre-operative TUS examination.

Table 1 lists and explains the names assigned to the different sonographic approaches to the lung parenchyma employed in the study.

From July 2018 to September 2019, 131 consecutive patients (41 women and 90 men, mean age 67.7  ± 8.9 years) with lung nodule and scheduled for surgery in our Unit of Thoracic Surgery, Fondazione Casa Sollievo della Sofferenza Hospital (San Giovanni Rotondo, Foggia, Italy) have been enrolled.

The inclusion criteria were: (1) age > 18 years; (2) a single pulmonary lesion indicated for VATS or open thoracotomy and (3) no contraindications for surgery. The exclusion criteria were: (1) a prolonged prothrombin time (PT-INR) >1.5 or a platelet count <30,000; (2) uncontrolled systemic hypertension (i.e., systolic blood pressure >140 mmHg); (3) recent myocardial infarction or unstable angina; (4) impossibility to tolerate single lung ventilation; (5) uncooperative behavior; (6) severe neurologic problems and (7) pregnancy. Before elective surgery, all patients underwent contrast-enhanced computed-tomography (CECT) and/or positron emission tomography with 2-deoxy-2-[fluorine-18]fluoro-D-glucose integrated with computed tomography (^18^F-FDG PET/CT) scan, according to the current diagnostic and staging protocol for lung cancer [1,17,18]. A pre-operative Transthoracic Ultrasound (TUS) investigation was also carried out.

The study followed the amended Declaration of Helsinki and the protocol was approved by the institutional review board of Fondazione Casa Sollievo della Sofferenza Hospital (TACE-CSS, n 106/2018, Approved date: 22 June 2018). All the enrolled patients were asked to sign a written informed consent for all the procedures included in the study. 

### 2.1. Pre-Operative Chest CT and Transthoracic Ultrasound (TUS)

All patients underwent pre-operative Chest CT (Toshiba, Tokyo, Japan) with the following protocol: tube voltage, 120 kVp; standard tube current, 60–120 mAs; slice thickness, 0.5 mm; reconstruction interval, 0.5–1.0 mm. CT images were acquired at full inspiration, with the patient in the supine position, before and after IV injection of the non-ionic contrast medium Iopamiro 370 mg/mL (Bracco, Milan, Italy). “Pulmonary nodules” were defined as single well circumscribed radiographic opacities surrounded by unaltered aerated lung. Pre-operative CT scan was used to define the size and the localization of the nodule. “Size” was defined as the mean between the maximum and minimum diameter in mm of the nodule on axial CT scan. Nodules were termed “peripheral” if they were located within 3 cm from the parietal pleural surface (i.e., in the outer third of the lung) and “central” if they were located in the inner two thirds of the lung. Location was defined on the basis of the affected right or left pulmonary lobe.

Pre-operative TUS investigation was carried out by an Esaote MyLab-9 scanner (Esaote-Biomedica, Genoa, Italy), equipped with a multi-frequency convex probe (2–8 MHz) and a multi-frequency linear probe (8–12.5 MHz). For the ultrasonographic examinations we used the following machine setting: depth varying between 70 and 140 mm, time gain compensation (TGC) between 40 and 50%, electronic imaging focus on the pleural line and use of tissue harmonics. The exam was focused on the localization of lung nodules that were previously assessed on Chest CT. “Lung nodules” were defined as subpleural consolidated areas interrupting the overlying pleural line echogenicity [10]. Their morphology was categorized as follows: according the shape in “regular” (i.e., round or oval) or “irregular” (i.e., polygonal, indefinite), according the margins in “well defined” or “jagged” and according the sonographic pattern in hypoechoic (i.e., blacker) or hyperechoic (i.e., brighter). The presence or absence of inner hyperechoic striae and/or spots was also recorded.

### 2.2. Surgical Techniques: Video-Assisted Thoracoscopic Surgery (VATS) and Open Thoracotomy

Both the procedures, open surgery or VATS, were performed by two dedicated thoracic surgeons with more than 5 years of experience. All the patients were placed in the lateral decubitus position under general anesthesia with selective one-lung ventilation through double-lumen endotracheal intubation. In addition, patients underwent ultrasound-guided fascial blocks of the chest wall (i.e., erector spinae plane block or serratus anterior plane block) using long-lasting local anesthetics in order to reduce post-operative pain.

VATS was performed using a three-port non-rib-spreading technique, according to Hansen et al. [19]. A 4 cm utility incision was placed at the fourth or fifth intercostal space in the midaxillary-line and the retraction of the tissues was achieved with a wound protector providing 360° of circumferential hands-free and atraumatic retraction. Two further thoracoscopic port were placed at the seventh or eighth intercostal space. The first port, with a size of 1 to 1.5 cm, was placed on the anterior axillary line for the introduction of a 10 mm 30° camera; the second port, of approximately 1 to 1.5 cm, was placed on the posterior axillary line. The surgeon then tried to localize the nodule by one-finger palpation.

Lateral muscle-sparing open thoracotomy was performed in the fifth space using the Noirclerc technique [20,21]. A muscle-sparing axillary thoracotomy was performed in some patients in order to obtain a better cosmetic result [22]. The section of the intercostal nerves was performed on the superior margin of the inferior rib in order to preserve the intercostal vascular-nervous bundle. The ribs were slowly spread through two retractors. The first retractor was placed parallel to the ribs, acting as a rib spreader; the second retractor was placed perpendicular to the ribs, retracting the preserved latissimum dorsi muscle anteriorly and inferiorly to enhance exposure. This allowed two hands to be placed in the chest and the lung was carefully bi-manually palpated.

### 2.3. Intraoperative Lung Ultrasound (ILU) Examination

The US processor used for ILU examination was the My Lab 25 GOLD (Esaote, Genova). A sterile intracavitary laparoscope probe with 10-mm diameter, 38 cm length and a flexible tip (LP 4–13, ± up/down 90°; right/left 90°) equipped with a linear array transducer with frequencies ranging from 8 to 12 MHz was introduced through one of the VATS ports or thoracotomy entry to the thoracic cavity. A setting for superficial tissue with tissue harmonics, gain <50%, and electronic focusing at the interface level was used. The lung under examination was inflated in order to avoid complications due to post-operatory pulmonary re-expansion. A partial desufflation of the lung was obtained by the application of pressure on the visceral pleura using the probe, in order to eliminate the air around the affected lung and improve the identification of deeper nodules. During the examination, the probe was positioned perpendicularly to the pulmonary surface. A warm sterile saline was used to improve surface contact. No change in the standardized surgical procedures was determined by the accommodation of the probe into the lung. A 10–15 min longer operative time was required by both the open thoracotomy and the VATS procedure with ILU compared to the surgery without ILU.

Localization and ILU pattern of the nodule/s were recorded. The nodules were classified according to their echogenicity in hypoechoic (i.e., blacker) or hyperechoic (i.e., brighter), according to their shape in “regular” (i.e., round or oval) or “irregular” (i.e., polygonal, indefinite) and according to their margins in “well defined” or “jagged”. The presence or absence of inner hyperechoic striae and/or spots was assessed. “Size” was defined as the mean between the maximum and minimum diameter of the nodule in mm on the ILU scan.

## 3. Statistical Analysis

Data were presented as means and standard deviations (mean ± SD) for continuous variables, and in number and percentage (*n*, %) for categorical variables. Accuracy of ILU or digital palpation for the detection of pulmonary nodules was calculated as rate of detected lesions in percentage. ILU findings were divided into three groups according their final diagnosis (primitive lung benign lesions, primitive lung malignant lesions and lung metastasis) and compared each other. ANOVA and unpaired Student’s t-test were used to assess the differences between groups. Fischer’s exact test was used to assess the differences between ILU and TUS examination. A *p* < 0.05 was considered as statistically significant.

## 4. Results

One nodule to be biopsied was identified by the pre-operative Chest CT in all the patients. Among 131 lung nodules, 54/131 (41.22%) were centrally located, 77/131 (58.78%) were peripheral. In details, 24/131 (18.32%) were located in the right superior lobe, 41/131 (31.30%) in the left superior lobe, 34/131 (25.95%) in the right inferior lobe, 21/131 (16.03%) in the left inferior lobe, and 11/131 (8.40%) in the middle lobe. The average diameter of the nodules measured on pre-operative Chest CT was 12.50 ± 2.19 mm. Patients’ demographic characteristics and lesions characteristics on pre-operative Chest CT are summarized in Table 2.

**Figure 1:**
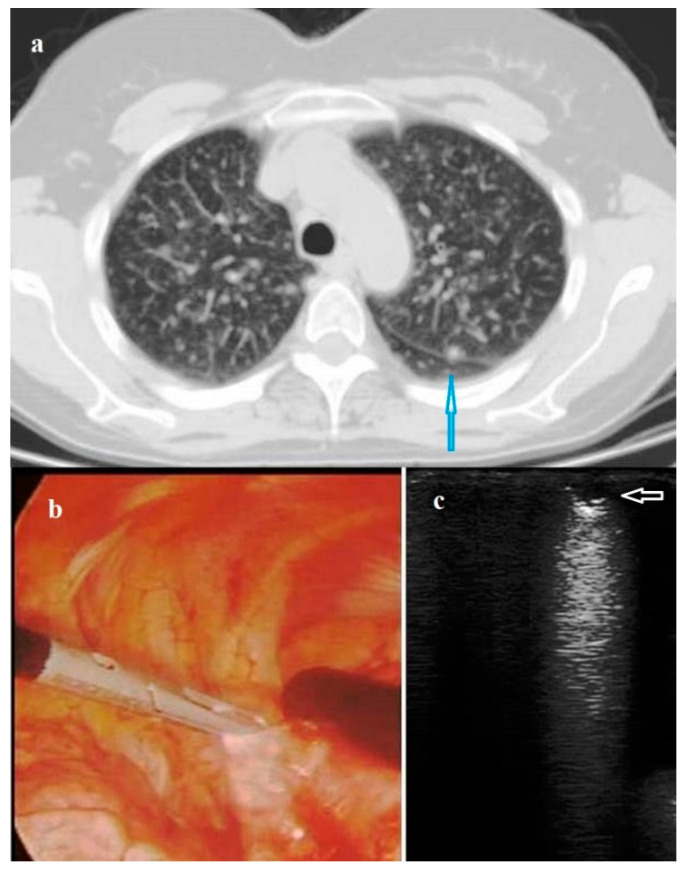
(**a**) Axial Chest Computed Tomography (CT) scan showing a not subpleural micronodule (blue arrow) in the left lung (maximum diameter 7.00 mm) not visualized during surgery nor identified by manual palpation. (**b**) Photograph showing the intracavitary laparoscope probe (8–12 MHz) placed on the left lung during Video-Assisted Thoracoscopic Surgery (VATS). (**c**) Intraoperative lung ultrasound during VATS (VAT-US) showing the pulmonary nodule (white arrow) with a hypoechoic US pattern, regular rounded shape and well-defined margins. The final Histological diagnosis was pulmonary Langerhans cell histiocytosis X.

VATS was performed in 49/131 (37.40%) patients, while open thoracotomy in 82/131 (62.60%) patients. 124/131 nodules (94.66%) were identified at digital palpation. The visualization of nodules was allowed by ILU in all the cases (100.00%). In addition, it was possible to detect by ILU also two CT-detected nodules (maximum diameter 9.00 mm and 7.00 mm, respectively) not visualized during surgery nor identified by manual palpation (Figure 1). The final histological diagnoses of the two nodules were hamartochondroma and histiocytosis x, respectively.

The average diameter of the nodules measured at ILU was 12.72 ± 2.27 mm. The actual histological margins of the lesions in the operating samples resulted to be corresponding to that assessed at ILU in 125/131 (95.42%) cases. No associated complications have been reported with ILU employment. The seven nodules that could not be identified by the digital palpation were proved to have statistically lower dimensions on CT scan compared to palpable nodules (9.92 ± 1.28 mm vs 12.63 ± 2.21 mm; *p* = 0.002). Three of them were centrally located, while the other four were peripheral but not subpleural.

With reference to the final diagnosis, 94/131 (71.76%) of cases resulted primitive pulmonary lesions, while 37/131 (28.24%) were metastatic lesions. Among the primitive nodules, 34 (25.95% of the total) had benign histology, and 60 (45.80% of the total) were malignant. Among the benign lesions, histological diagnoses were: hamartoma (four cases), hamartochondroma (two cases), fibrolipoma (four cases), organizing pneumonia (six cases), granuloma (two cases), lung abscess (two cases), histiocytosis x (three cases), lung carcinoid (three cases), fibrotic nodules in UIP (eight cases). Malignant nodules were diagnosed as follows: adenocarcinoma (38 cases), squamous cell carcinoma (14 cases), small cell lung cancer (six cases), large-cell undifferentiated carcinoma (one case), pulmonary lymphoma (one case). Among metastases, nine were from colon cancer, eight from melanoma, three from breast cancer, one from gastric cancer, two from liver (hepatocarcinoma), five from kidney (clear cell papillary renal carcinoma), four from urothelium, three from sarcoma, one from larynx, one from oral cable.

**Figure 2:**
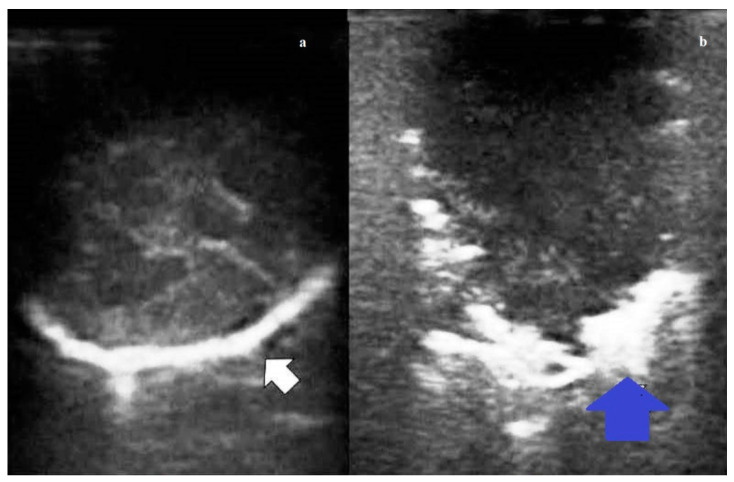
(**a**) Intraoperative lung ultrasound (ILU) scan showing a hyperechoic pulmonary nodule (white arrow) with a regular rounded shape and well-defined margin. The final histological diagnosis was pulmonary hamartoma. (**b**) Intraoperative lung ultrasound (ILU) scan showing a hypoechoic nodule (blue arrow) with irregular shape and jagged margin. The final histological diagnosis was pulmonary adenocarcinoma.

According to the ILU pattern, 125 (95.42%) nodules were classified as hypoechoic and six (4.58%) as hyperechoic. Among the hypoechoic nodules, 37 (28.24%) had intralesional hyperechoic spots. Benign nodules were in the vast majority of cases (94.12%) hypoechoic, with internal hyperechoic spots only in 38.24% of cases; they had regular rounded shape in all cases, and no jagged margins in 91.18% of cases. Malignant nodules were mostly hypoechoic (96.67% of cases) with jagged margins (68.33%). Internal hyperechoic spots were found only in the 21.65% of cases. A regular rounded shape was observed in roughly the half (53.33%) of cases (Figure 2). Metastases were mostly hypoechoic (94.59%), with jagged margins (100.00%). Internal hyperechoic spots were seldom assessed (29.73%) (Table 3).

**Figure 3:**
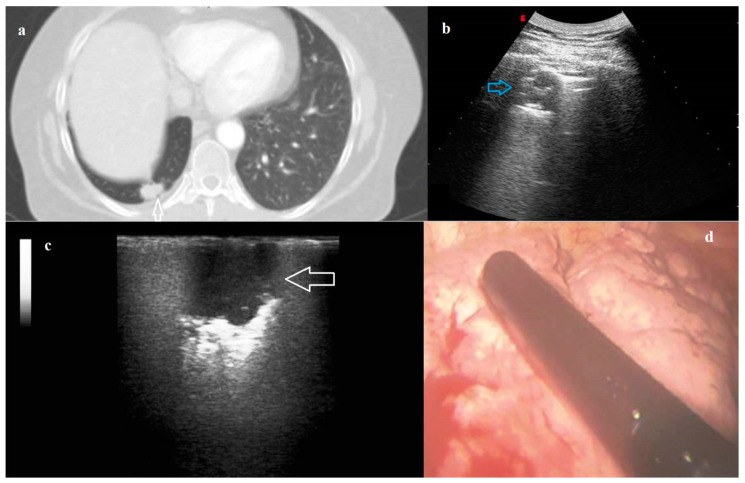
(**a**) Axial computed tomography (CT) scan showing a pulmonary nodule (white arrow) in the right lung. (**b**) Transthoracic ultrasound (TUS) scan (convex probe, 5 MHz) showing the right subpleural nodule adhering to pleural surface (blue arrow). (**c**) Intraoperative lung ultrasound during Video-Assisted Thoracoscopic Surgery (VATS-US) showing the pulmonary nodule (white arrow) with a hypoechoic pattern and jagged margins. (**d**) Photograph showing the intracavitary laparoscope probe (8–12 MHz) placed on the right lung during Video-Assisted Thoracoscopic Surgery (VATS). The final histological diagnosis was pulmonary adenocarcinoma.

The identification of 40/131 (30.53%) nodules was allowed by TUS. All the nodules identified on TUS were peripherally located and adherent to the parietal pleural surface. The sonographic pattern of nodules on TUS was characterized by a greater appearance of jagged margins (82.50% versus 52.50%, *p* = 0.004), hyperechoic pattern (32.50% versus 10.00%, *p* = 0.01) and hyperechoic striae (40.00% versus 12.50%, *p* = 0.005) compared to ILU (Figure 3). On TUS, we did not record any statistically significant differences in terms of shape, margins and echogenicity between primary benign and malignant lung nodules and metastasis (Table 4).

## 5. Discussion

Intraoperative lung ultrasound (ILU) has been suggested to be an effective and safe method for improving the localization of difficult to visualize pulmonary nodules during surgery by results of the present study. In addition, for the first time, descriptive and illustrative data derived from the systematic application of ILU during thoracic surgery (VATS and open thoracotomy) in a cohort of patients with lung nodule have been presented, allowing to enhance the knowledge on the direct ultrasound examination of the pulmonary district.

Ultrasound (US) have been shown to be effective in guiding various interventional procedures in the thoracic district, providing real-time imaging without any radiation hazard. Transthoracic ultrasound (TUS) can be used as a guide for collecting samples of pleural fluid for chemicophysical, microbiological and cytological analyses or for evacuation thoracentesis. In addition, TUS is widely and successfully utilized as a guide for transthoracic needle biopsy (US-TNB) of peripheral lung lesions adhering to the pleural surface [11,12]. Endobronchial ultrasound-guided transbronchial needle aspiration (EBUS-TBNA) has become an important procedure for diagnosing lung cancer, particularly when the evaluation of mediastinal lymph nodes is required [23]. The decreased rate of complications, together with the increased accuracy of procedures performed under US guide, are obvious benefits for operators and patient alike for all interventional applications. Similarly, a useful application during surgical procedures on the lung can be supposed for ILU.

VATS is a minimally invasive thoracic surgery that has been shown to be particularly useful in patients who are debilitated or have marginal pulmonary reserve. Indeed, the avoidance of a thoracotomy incision was associated with less postoperative pain and morbidity and earlier recovery compared to open thoracotomy [5]. However, bimanual lung palpation is frequently prevented during traditional VATS techniques. This makes the localization of non-subpleural lung nodules more difficult. On the contrary, open thoracotomy techniques, allowing for bimanual palpation of the lung parenchyma, have been proven to have the advantage of easier lung nodules localization. Failure to localize small and relatively deep pulmonary nodules during VATS often leads to conversion to open thoracotomy. The present study was not designed to demonstrate the superiority of one surgical technique over the other, but to demonstrate the sensitivity of ILU in helping to detect lung nodules during both the surgical techniques.

VATS-US has been showed to be superior in the detection of pulmonary micro-nodules compared with traditional VATS or finger palpation by available studies on this novel technique [13,24,25]. The high detection rate of pulmonary nodules using ILU was confirmed also in the present study, with 100.00% of lung nodules assessed by ILU compared to about 95% by finger palpation during both the surgical procedures. Most of the lung nodules included in this study (about 96%) were sized <2 cm and the dimension of the seven nodules that escape the detection by digital palpation was statistically lower compared to that of palpable nodules. Indeed, ILU was reported to be a useful technique in identifying even very small pulmonary nodules (<2–4 mm) by some authors [26,27]. Furthermore, ILU was demonstrated to be able to detect nodules deeply situated in the lung parenchyma [26]. Accordingly, 54 (41%) lung nodules identified by ILU in this study were centrally located. Three of the nodules that could not be identified by the digital palpation in the current experience were centrally located; the remaining other four were peripheral but not subpleural. The detection of central nodules was made possible by the partial desufflation of the lung, together with the pressure applied on the visceral pleura with the probe.

In some studies ILU has been shown to be able even to identify small nodules not identified before by pre-operative Chest-CT [14,16]. This evidence was confirmed also by our previous experiences [9,13,28]. However, in the present study no time was spent in searching for other nodules eventually not previously identified on the preoperative CT scan because the main goal was focused in finding and assessing the histological nature of the nodule of interest and not of other nodules.

**Figure 4:**
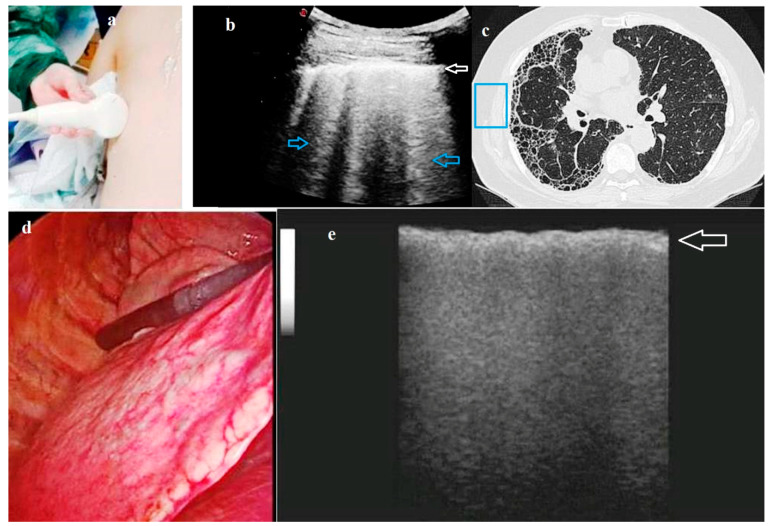
A case of Usual Interstitial Pneumonia (UIP) fibrosis (histological diagnosis). (**a**) Photograph taken during the pre-operatory Transthoracic Ultrasound (TUS) examination showing a multi-frequency convex probe (2–8 MHz) placed on the posterior chest wall. (**b**) Pre-operatory TUS scan (convex probe, 5 MHz) showing an increased thickness of the hyperechoic pleural line (white arrow) and an increased number of B lines below it (blue arrows). (**c**) High-Resolution Computed Tomography (HRCT) scan of the same patient showing a pattern of fibrosis. The blue box enhances the area corresponding to the TUS scan showed in b). (**d**) Image of the pulmonary parenchyma during video-assisted thoracoscopic surgery (VATS). (**e**) Intraoperatory lung ultrasound (ILU) scan (linear probe, 12 MHz) showing an irregular thickening (white arrow) of the interface line between lung and the ultrasound probe (i.e., the intraoperatory “pleural line”) with no artifact below it.

A systematic evaluation of the lung parenchyma without artifacts due to the elevated difference in acoustic impedance encountered by the ultrasound beam at the interface between the aerated lung parenchyma and the overlying soft tissues of the chest wall can be theoretically made possible by the ILU approach. As a confirmation, B-lines artifacts have been shown to be absent in ILU scans even in patients with pulmonary fibrosis, despite these ultrasound findings were observable at TUS examination in our previous experience [29] (Figure 4). During open thoracotomy, in which the ribs are spread, even the obstacle constituted by these bony structures is eliminated. As the probe is placed directly on the lung surface, high-frequency transducers can be used, affording much better resolution than the low-frequency transducers used for TUS.

In previously published data, nodules characteristics at ILU have been shown to correlate with probability of malignancy [16,24,25]. Similarly, in the present study a malignant lesion seemed to be indicated more by an irregular shape and the presence of jagged margins rather than by well-defined rounded margins. This morphological differentiation was not possible by TUS examination, where the more air-filled periphery of the nodule is known to create ultrasound artifacts at the interface with the healthy aerated lung parenchyma resulting in irregular and blurred deep margins. On the contrary, the echographic pattern (i.e., hypoechoic or hyperechoic appearance) and the presence of hyperechoic spots within the lesion at ILU were not shown to present any correlation in terms of histology between benign and malignant lesions. On TUS evaluation, a hyperechoic pattern and the presence of hyperechoic striae have been observed more than on ILU, suggesting that these images are probably the result of the difference in acoustic impedance that ultrasound comes to find at the interface between the aired lung content and soft tissues of the chest wall. Another possible explanation lies in the interposition of also few microns of air between the nodule and the probe due to an incomplete adherence of the lesion to the parietal pleura [30]. When the acoustic attenuation of the chest-wall structures is removed and the probe is directly placed on lung parenchyma (as during ILU examination), the reflection phenomena of the ultrasound beam are reduced and a hypoechoic appearance for lung nodules results increased. A hyperechoic pattern and the presence of hyperechoic striae on ILU examination can be still explained by an inhomogeneous tissue composition between different areas within the nodule. This suggestion needs to be carefully evaluated and requires further study to be confirmed. Obviously, more detailed data on the morphology of pulmonary nodules are pre-operatively provided from CT and PET scans. However, despite the characterization of the lung nodules at ILU not having a strictly clinical value, it can help in better understanding the influences that the pulmonary air content and the elevated difference in acoustic impedance generated at the interface with the overlying soft tissues of the chest wall have in the transthoracic study of pulmonary diseases. This allows, at the same time, a more correct interpretation of ultrasound artifacts.

Anyhow, using the high-quality imaging of ILU guidance to identify small and deep nodules, a strong correlation was found between margins measured on ultrasound examination and the resection margins verified on histology. In particular, the margins identified with the ILU and the actual histological margins of the lesions were observed to correspond in about 95% of the operating samples in the current experience.

The main limit of our study relies in the small number of the enrolled patient. However, this limitation must be related to the novelty of ILU examination. Furthermore, our sample size was also larger than that characterizing other works employing the same intra-operatory technique [14,15,16,24,25]. Another possible limitation is that the ILU study of pulmonary nodules was shown to prolong the operative time by 10–15 min both in VATS and open thoracotomy. Taking this into account, in larger prospective randomized controlled trial (RCT) it could happen that anesthesia-related complications will be caused by ILU application in some cases or that the exam will demonstrates to be not cost effective. Despite these possibilities remains to be excluded, in the present study ILU has shown to be an extremely safe method for the detection of pulmonary nodules, with no associated post-operatory or anesthesia related complications.

## 6. Conclusions and Areas for Future Research

In conclusion, in our experience ILU performed during both VATS and open surgery has proved to be a reliable and safe method for the real-time detection of pulmonary nodules not previously assessed by digital palpation. This seemed to be useful especially for small nodules that, being centrally located or, in any case, not being in a subpleural position, can more easily escape the surgeon’s feel during manual palpation. Despite, as in the TUS approach, was not possible to find a specific echostructural pattern that correlates with the benign or malignant nature of the lesions by ILU, the intra-operatory technique, using a high-frequency linear probe (12 MHz), resulted in a clear definition of the margins of the lesion, that matched, in most cases, with the actual histological margins in the operating samples. In this perspective, the well-defined interface between the nodules and the surrounding parenchyma could help the surgeon in deciding the extension of pulmonary resection. In addition, the ILU study of pulmonary diseases may improve the knowledge on the sonographic study of the lung, allowing a more correct interpretation of ultrasound artifacts. ILU application in larger prospective randomized controlled trial (RCT) will definitely clarify the actual effectiveness and benefits of this novel technique in the detection of pulmonary nodules, simultaneously allowing the possible complications to be excluded. In particular, the assessment of the interface between the nodules and the parenchyma and the marking of the margins is a completely innovative intuition, which we hope can be confirmed and validated by future studies.

## Figures and Tables

**Table 1:** Table of nomenclature of the different sonographic approaches to the lung parenchyma.

Acronym	Meaning	Definition
TUS	Transthoracic Ultrasound	Sonographic examination of the lung achieved through the thorax
ILU	Intraoperative Lung Ultrasound	Sonographic examination of the lung performed during surgical procedures (VATS or open surgery) with an intracavitary laparoscope probe directly placed on lung parenchyma
VATS-US	Video-Assisted Thoracoscopic Surgery-Ultrasound	Sonographic examination performed during Video-Assisted Thoracoscopic Surgery (VATS)

**Table 2:** Patients’ demographic characteristics and lesions’ characteristics on pre-operative Chest CT.

Characteristics	*n* = 131	Min-Max or %
Age	67.7 ± 8.9	19–88
Sex		
Male	90	68.70%
Female	41	31.30%
Lesion Size on CT scan (mm)	12.50 ± 2.19	7.00–25.60
Central	54	41.22%
Peripheral	77	58.78%
Lesion location		
Right upper lobe	24	18.32%
Right middle lobe	11	8.40%
Right lower lobe	34	25.95%
Left upper lobe	41	31.30%
Left lower lobe	21	16.03%

**Table 3:** Ultrasound characteristics of nodules examined using ILU and their relationship to their pathologic nature.

Characteristics	Benign (*n* = 34)	Malignant (*n* = 60)	Metastasis (*n* = 37)	*p*-Value
Shape				
Regular	34 (100.00%)	32 (53.33%)	5 (13.51%)	<0.0001
Irregular	0 (0.00%)	28 (46.66%)	32 (86.49%)	<0.0001
Margins				
Well defined	31 (91.18%)	19 (31.67%)	0 (0.00%)	<0.0001
Jagged	3 (8.82%)	41 (68.33%)	37 (100.00%)	<0.0001
Echogenicity				
Hyperechoic	2 (5.88%)	2 (3.33%)	2 (5.41%)	0.82
Hypoechoic	32 (94.12%)	58 (96.67%)	35 (94.59%)	0.82
Hyperechoic spots	13 (38.24%)	13 (21.65%)	11 (29.73%)	0.23

**Table 4:** Ultrasound characteristics of nodules examined using TUS compared to those recorded at ILU and their relationship to the pathologic nature of the nodule.

Total (*n* = 40)				
	**TUS**	**ILU**		***p*-Value**
Shape				
Regular	12 (30.00)	19 (47.50)		0.11
Irregular	28 (70.00)	21 (52.50)		0.11
Margins				
Well defined	7 (17.50)	19 (47.50)		**0.004**
Jagged	33 (82.50)	21 (52.50)		**0.004**
Echogenicity				
Hyperechoic	13 (32.50)	4 (10.00)		**0.01**
Hypoechoic	27 (67.50)	36 (90.00)		**0.01**
Hyperechoic spots	16 (40.00)	5 (12.50)		**0.005**
**TUS** **(*n* = 40)**	**Benign** **(*n* = 14)**	**Malignant** **(*n* = 18)**	**Metastasis** **(*n* = 8)**	***p*-Value**
Shape				
Round	6 (42.86)	2 (11.11)	4 (50.00)	0.06
Irregular	8 (57.14)	16 (88.89)	4 (50.00)	0.06
Margins				
Well defined	5 (35.71)	1 (5.56)	1 (0.00)	0.08
Jagged	9 (64.29)	17 (94.44)	7 (100.00)	0.08
Echogenicity				
Hyperechoic	4 (28.57)	6 (33.33)	3 (37.50)	0.09
Hypoechoic	10 (71.43)	12 (66.67)	5 (62.50)	0.09
Hyperechoic spots	6 (42.86)	5 (27.78)	5 (62.50)	0.25

## Data Availability

The data presented in this study are available on request from the corresponding author. The data are not publicly available due to ethical reasons.
